# Data on acetic acid–methanol–methyl acetate–water mixture analysised by dual packed column Gas Chromatography

**DOI:** 10.1016/j.dib.2018.03.111

**Published:** 2018-03-29

**Authors:** Mallaiah Mekala, Venkat Reddy Goli

**Affiliations:** a Department of Chemical Engineering, BV Raju Institute of Technology, Hyderabad 502313, India; b Department of Chemical Engineering, National Institute of Technology, Warangal 506004, India

**Keywords:** Gas Chromatography, Esterification, Calibration, Retention time, Optimization

## Abstract

The composition of multicomponent determination by colorimetric titration is difficult. This complexity is easily overcome by using Gas Chromatography technique instead of wet method for multi-component mixture analysis. In Gas Chromatography, first the standard chart is prepared by using the known amount sample concentration as the reference. Once calibration chart is prepared the unknown sample concentration easily measured by using the standard chart. In the present study a standard calibration chart developed for the four component system of acetic acid–methanol–methyl acetate–water. The samples were taken at various concentrations of all components and different chromatograms obtained under various concentrations respectively. The method of optimization was first carried out to get the sharp peaks of individual components and binary pairs also. By using those conditions, the multi components concentrations were estimated. From the present results, the area under gas chromatogram is linearly varying with mole% of the components compared to mass%.

**Specifications Table**

**Table:** 

**Subject area**	Chemistry
**More specific subject area**	Analysis of compounds for multi-component mixture
**Type of data**	Table, text file, graph, figure
**How data was acquired**	Analytical method
**Data format**	Analyze
**Experimental factors**	–
**Experimental features**	*Determination of the individual component composition in the multi-component mixture.*
**Data accessibility**	*With this article*.

**Value of the data**

• The analysis of the multi-component mixture is carried by using the GC dual packed column useful to chemical as well as the pharmaceutical industry.
• A detailed calibration is given in this data article which should be useful to analysis of other mixtures.
• Analysis of the different mixtures for the research of reactive distillation, this method as well as data is useful.

## 1 Data

Methyl acetate is commonly produced from a liquid state reaction between acetic acid and methanol. The products are methyl acetate and water as shown in the following equation.

(1) CH3COOH+CH3OH↔CH3COOCH3+H2O

The concentrations of the multi-component mixture s measured by titrating the reaction mixture with standard sodium hydroxide to find the concentration of the un-reacted acetic acid concentration assumed that the reaction proceeds as per the stoichiometry [1], [2], [3], [4]. But when the reaction forms azeotropes, side reaction is taking place or undesired product forms, it is difficult to measure the concentrations at desired operating conditions [5], [6], [7], [8]. That means the concentration is not varying according to the stoichiometric reaction. So that the assumption is not applicable in that cases. So the alternate method to measure the concentration of the multi component mixture is use of instruments like Gas Chromatography, which gives accurate composition of the mixtures [9], [10], [11], [12], [13], [14].

The reaction between the acetic acid and methanol to produce methyl acetate and water in presence of the catalysts. The numbers of studies are available on this system in presence of the different liquid and solid catalysts [4], [7]. Many of the authors used the wet titration method to measure the concentration of the four component mixture. Some of the authors used the Gas Chromatography method and did not mention the standard calibration chart preparation. Many of the authors given the specifications of the column, detectors and operating conditions but did not mentioned the preparation the reference samples to measure the unknown samples concentrations.

Hence we have taken this problem to give a detailed procedure for the analysis of the multicomponent mixture samples by using the dual packed column with TCD detector. The method of optimization for getting the sharp peaks, calibration chart and measuring the concentration of the samples were described to give the clear explanation for the readers.

## 2 Experimental design, materials and methods

### 2.1 Chemicals

Methanol (purity = 99% w/w) and Acetic acid (purity = 99.95%w/w) supplied by SD Fine Chemicals Ltd. (Mumbai, India).

### 2.2 Gas Chromatography

**Fig. 1:**
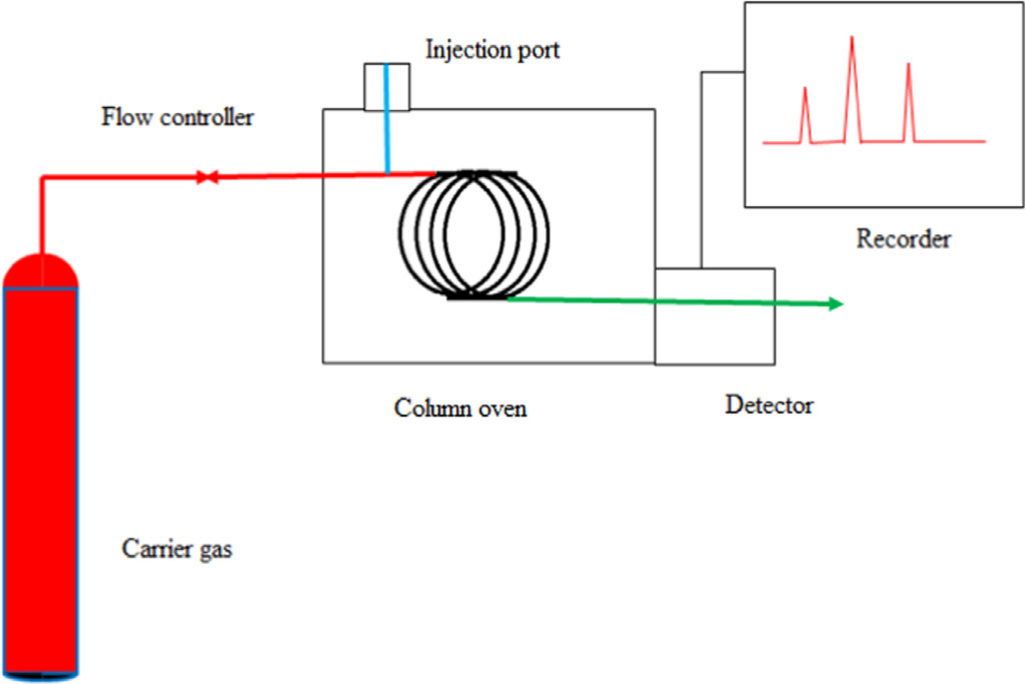
Schematic diagram of Gas Chromatography.

Gas Chromatography is a process for separating and analyzing the compounds that can be vaporized without decomposition. The schematic diagram of Gas Chromatography is shown in Fig. 1. It consists of a long spiral column, an inert gas cylinder, sample injection port, a detector at outlet which is connected to a computer for data acquisition.

When a liquid sample is injected into the injection port, it gets vaporized and the vapor is carried by the carrier gas through the column to the detector. The detector passes the signals to the recorder which displays the chromatogram on a computer. The thermal conductivity detector can detect all compounds except the carrier gas itself. The metallic TCD filament is heated by the application of current. As sample compounds pass the filament, the filament temperature increases, because the thermal conductivity of the sample compounds is less than that of the carrier gas. The filament temperature changes affect its resistance; the resistance is measured and produces a chromatogram. The TCD sensitivity is proportional to the difference in thermal conductivities between the sample and the carrier gas.

**Fig. 2:**
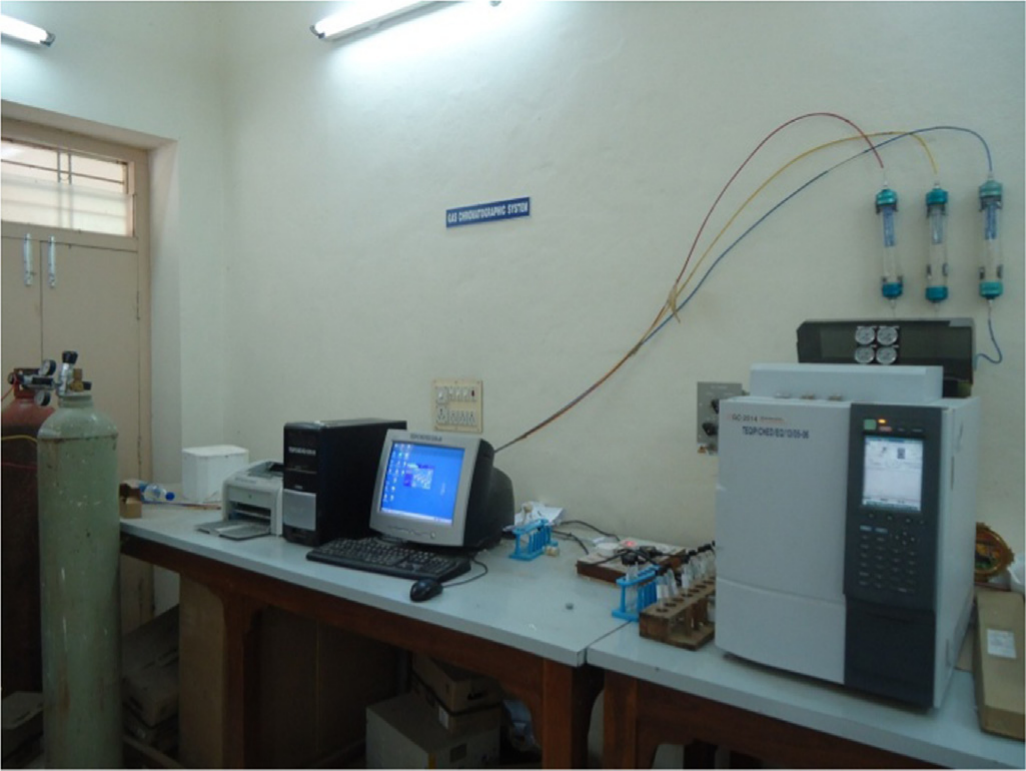
The laboratory Gas Chromatography instrument for the analysis of the chemical components.

Fig. 2 shows the laboratory Gas Chromatography instrument used for the analysis of the acetic acid, methanol, methyl acetate and water. It was equipped with an injection port, packed/capillary columns and FID/TCD detector. The carrier gas was the hydrogen. The inlet and outlet pressures of the hydrogen cylinder are adjusted by the control valves. The hydrogen carrier gas is allowed to the GC instrument at a constant pressure by using the pressure controller valve.

**Fig. 3:**
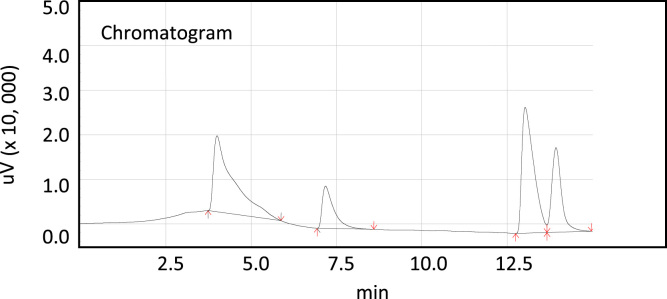
Characteristic peaks of a Gas Chromatograph for the four component system of Acetic acid–Methanol–Methyl Acetate–Water. Approximate peak times are Water: 4.1 min, Methanol: 7.2 min, Methyl Acetate: 12.8 min and Acetic Acid: 13.5 min.

**Fig. 4:**
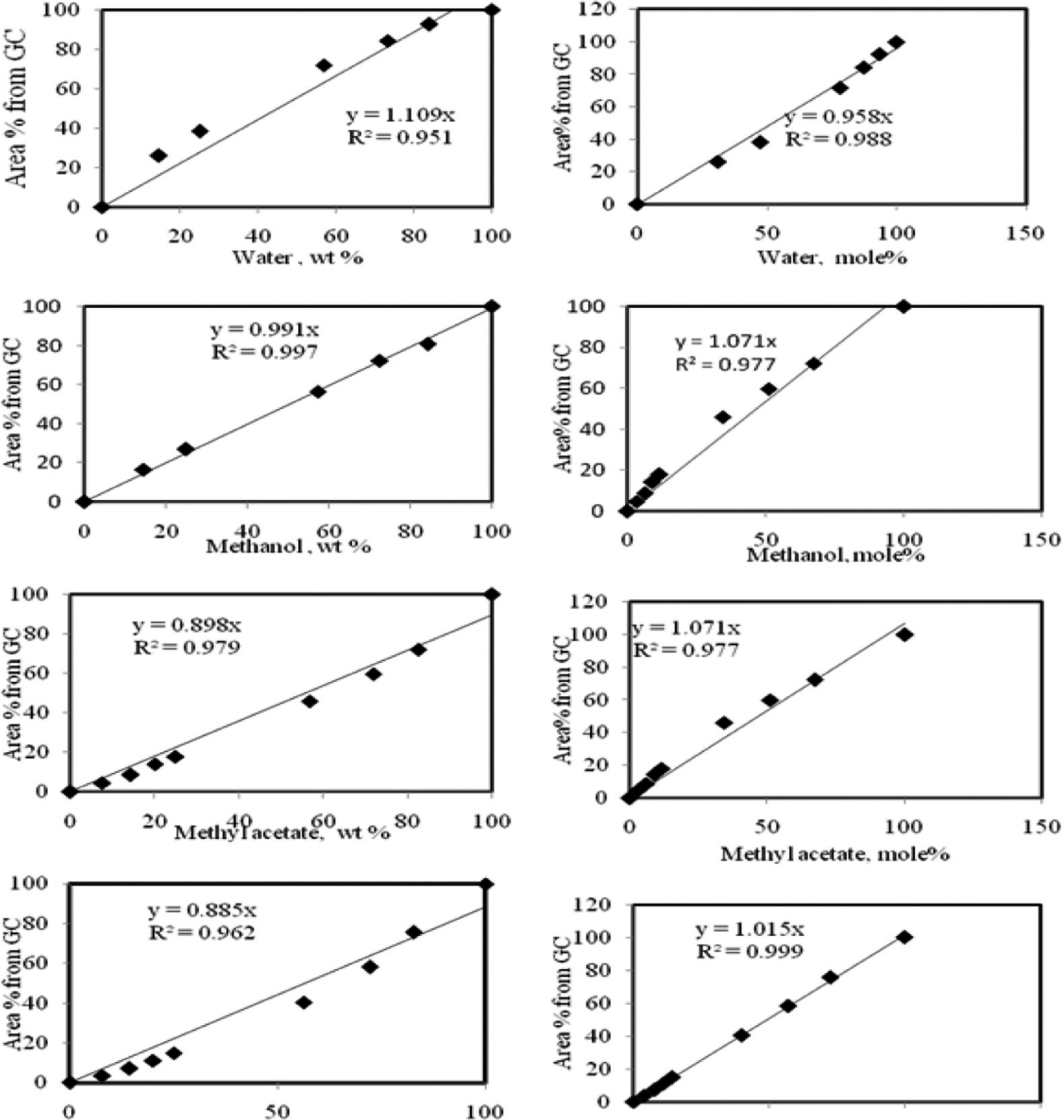
Calibration charts for the components acetic acid, methyl acetate, methanol and water based on area % vs wt%(left side) and area% vs mole%(right side) from GC analysis.

### 2.3 Optimization program

A micro litre of every sample was injected into the Gas Chromatography instrument for the analysis. The samples were analyzed using Gas Chromatography (GC-2014 ATF, Shimadzu, Japan.) equipped with a thermal conductivity detector. Porapak-Q (2 m length and 3.17 mm id) packed column was used to analyze the sample. High purity hydrogen gas was used as a carrier gas at a flow rate of 30.0 ml/min. The oven temperature was programmed at 323 K for 1 min and then raised from an initial value of 323 K to 443 K at a ramp rate of 10 K/min and was held at 443 K for 2 min. The detector temperature was maintained at 473 K.

### 2.4 GC parameter calibrations

It is necessary to determine which parameters are suitable for the operation of Gas Chromatograph. These parameters are related to the peaks obtained in a chromatogram for the mixture of compositions needs to analyze. The main parameters are flow rate of carrier gas, temperature of GC oven and range of detectors.

Volumetric flow rate of the carrier gas is controlled in GC software by setting pressure in the packed column. The flow rate of carrier gas depends on the column types. The correct settings for carrier gas flow rate as per the manufacturer's information for dual packed column of 3.17 mm id and 2 m length.

The next parameter that needs to be set is oven temperature. This value is usually limited upwards by column specification and downwards by analysis speed. Generally, when the temperature is high, the response of the system is fast but the separation of the peaks is poor. So that in this case, does not use the fixed temperature for the analysis but rather a ramped temperature. By applying the ramp input, the temperature increased or decreased during the experiment in order to get the better separation of the peaks. Finally the range of the detector should be set. The range of the detector is determined based on scale of power of the 10(10^range^). The detector should work in order to obtain a good peaks and to avoid the overload. The range of the detector is used a constant value of 3 throughout the experimental analysis.

### 2.5 Calibration

In order to calibrate the Thermal Conductivity Detector (TCD) for the analysis, it is necessary to prepare the standard solutions of known mixtures with different compositions. These compositions are chosen to get the real concentrations that are expected in the reactor may exist at any time. The different sets of the experiments performed for the preparation of the standard calibration.

All the samples are weighed using electronic weighing balance. The standards are prepared on the mass basis. From the mass basis, all the samples are converted to molar basis. All the components are prepared with precision scale and with the pure components to avoid contamination. Table 1, shows the standard samples which are prepared and used for the calibration of the GC.

**Table 1:** Gas Chromatography calibration standards.

**Standard**	**Mass fraction**				**Mole fraction**			
	**Water**	**Methanol**	**Methyl acetate**	**Acetic acid**	**Water**	**Methanol**	**Methyl acetate**	**Acetic acid**
1	0.142857	0.285714	0.285714	0.285714	0.311382	0.350305	0.151483	0.186829
2	0.25	0.25	0.25	0.25	0.474892	0.267127	0.115514	0.142468
3	0.571429	0.142857	0.142857	0.142857	0.783431	0.11017	0.047641	0.058757
4	0.727273	0.090909	0.090909	0.090909	0.878566	0.061774	0.026713	0.032946
5	0.842105	0.052632	0.052632	0.052632	0.935358	0.032884	0.01422	0.017538
6	0.285714	0.142857	0.285714	0.285714	0.548097	0.154152	0.133321	0.164429
7	0.25	0.25	0.25	0.25	0.474892	0.267127	0.115514	0.142468
8	0.142857	0.571429	0.142857	0.142857	0.263627	0.59316	0.064125	0.079088
9	0.090909	0.727273	0.090909	0.090909	0.165474	0.744633	0.04025	0.049642
10	0.052632	0.842105	0.052632	0.052632	0.094847	0.853627	0.023071	0.028454
11	0.307692	0.307692	0.076923	0.307692	0.519937	0.292464	0.031618	0.155981
12	0.285714	0.285714	0.142857	0.285714	0.504001	0.283501	0.061297	0.1512
13	0.142857	0.142857	0.571429	0.142857	0.352675	0.19838	0.343143	0.105802
14	0.090909	0.090909	0.727273	0.090909	0.262574	0.147698	0.510955	0.078772
15	0.052632	0.052632	0.842105	0.052632	0.17378	0.097751	0.676334	0.052134
16	0.285714	0.285714	0.285714	0.142857	0.511315	0.287614	0.124374	0.076697
17	0.25	0.25	0.25	0.25	0.474892	0.267127	0.115514	0.142468
18	0.142857	0.142857	0.142857	0.571429	0.332696	0.187142	0.080926	0.399236
19	0.090909	0.090909	0.090909	0.727273	0.23777	0.133746	0.057836	0.570648
20	0.052632	0.052632	0.052632	0.842105	0.151383	0.085153	0.036823	0.72664

**Fig. A1:**
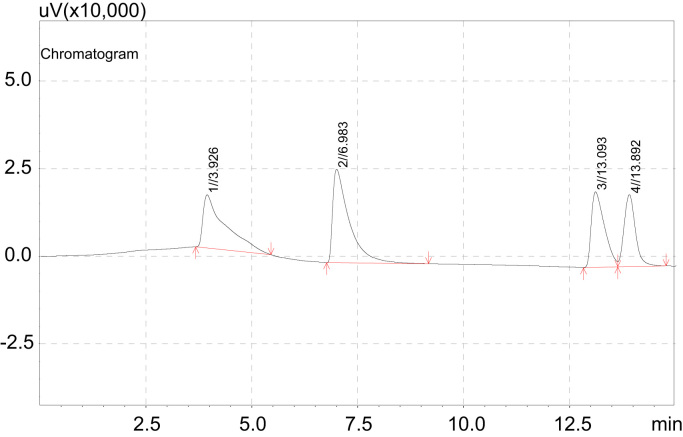
Sample standard.

**Fig. A2:**
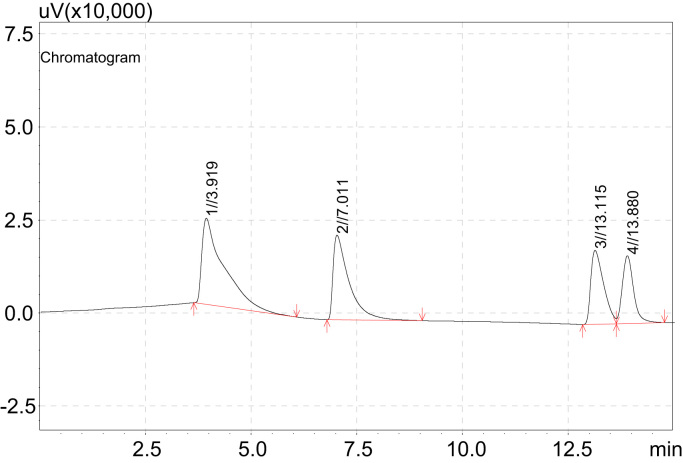
Sample standard.

**Fig.A3:**
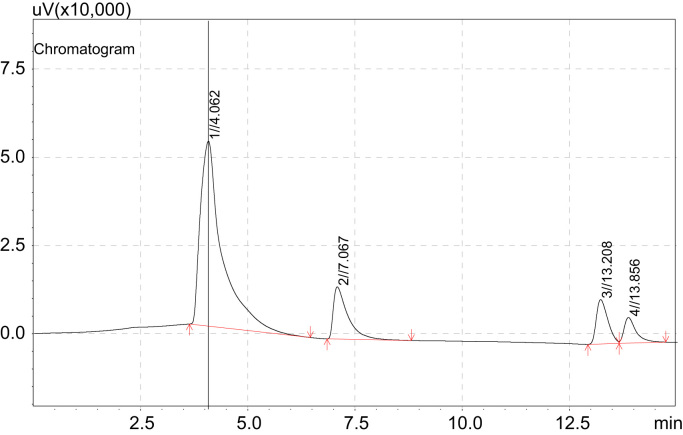
Sample standard.

**Fig. A4:**
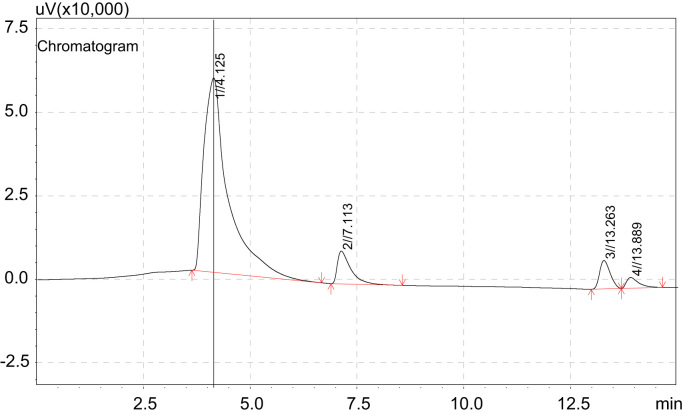
Sample standard.

**Fig. A5:**
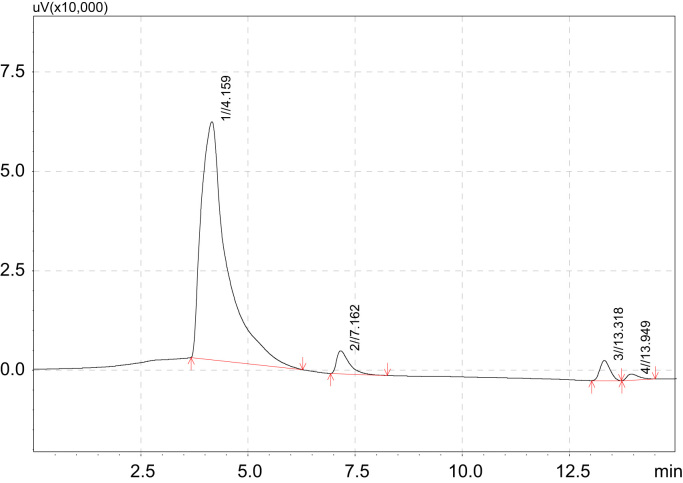
Sample standard.

**Fig. A6:**
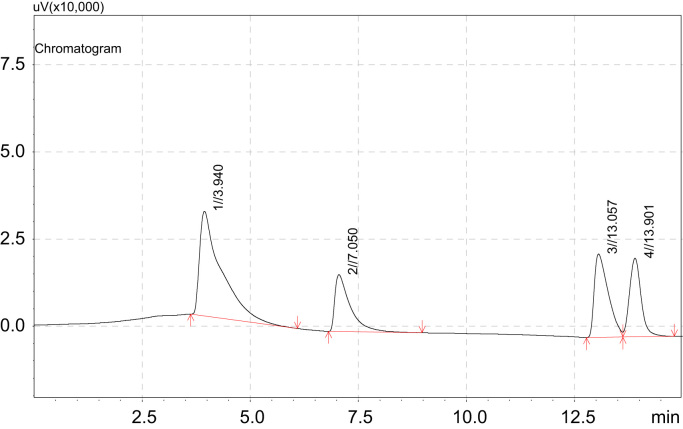
Sample standard.

**Fig. A7:**
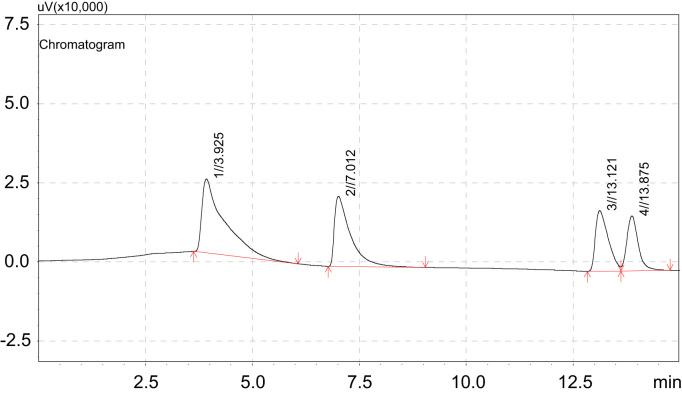
Sample standard.

**Fig. A8:**
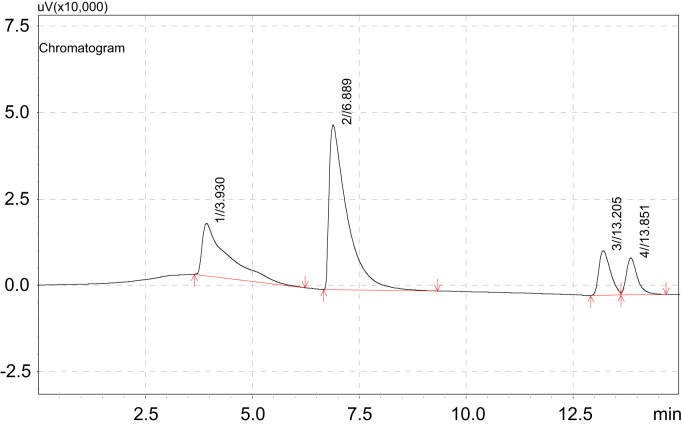
Sample standard.

**Fig. A9:**
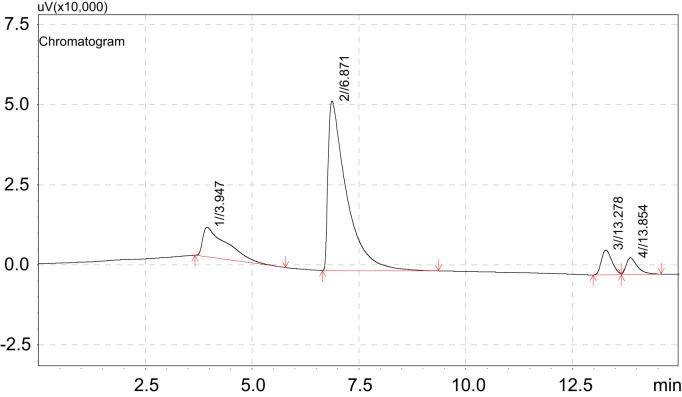
Sample standard.

**Fig.A10:**
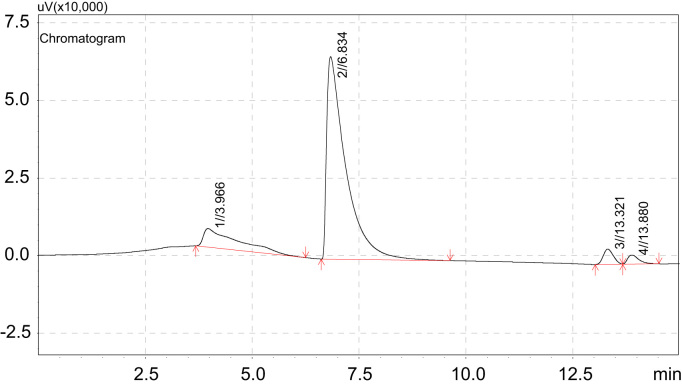
Sample standard.

**Fig. A11:**
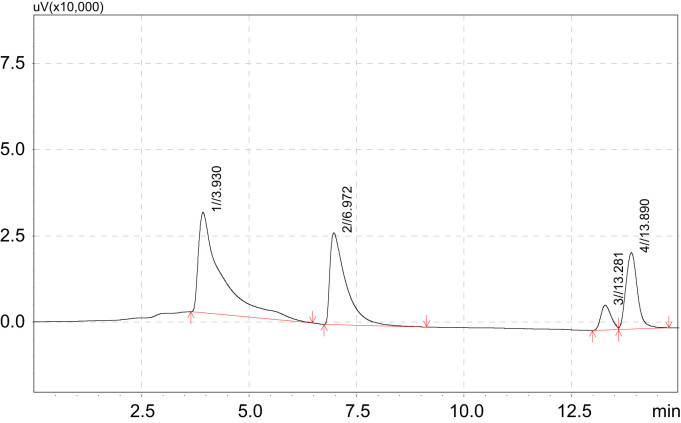
Sample standard.

**Fig. A12:**
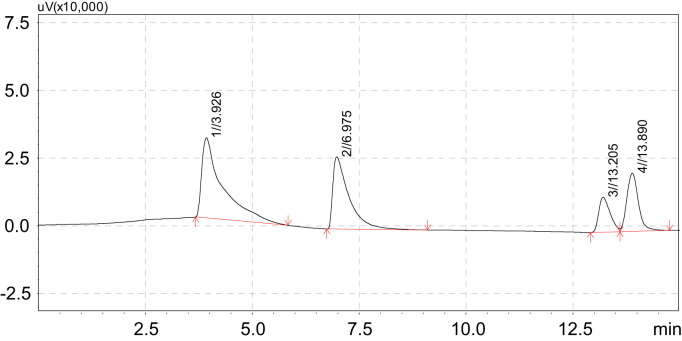
Sample standard.

**Fig. A13:**
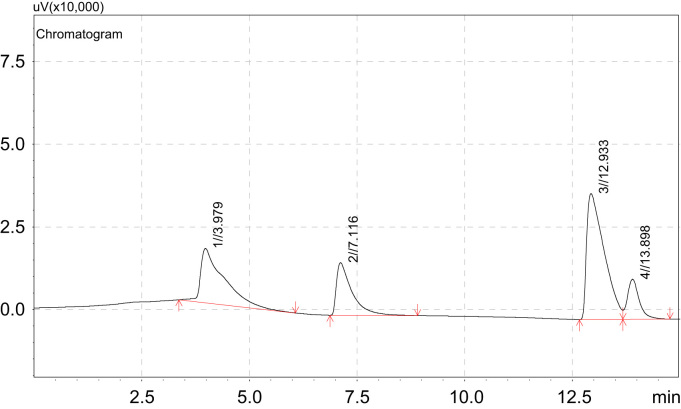
Sample standard.

**Fig. A14:**
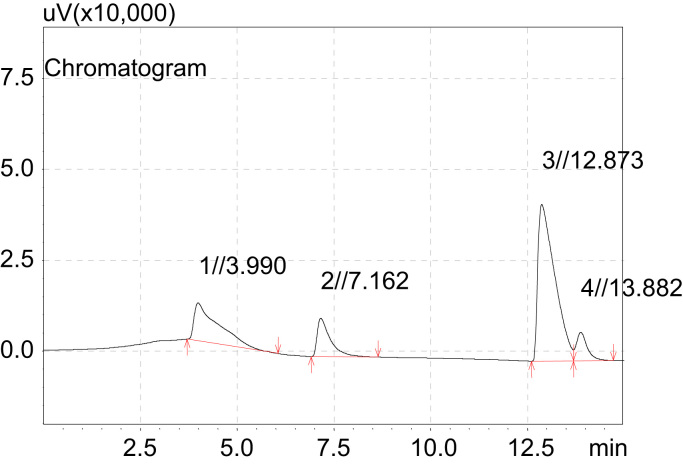
Sample standard.

**Fig. A15:**
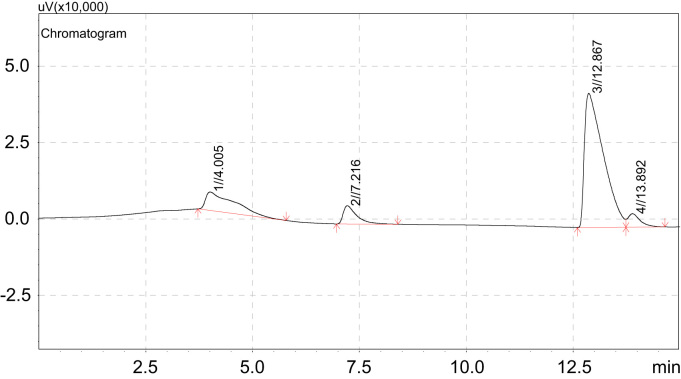
Sample standard.

**Fig. A16:**
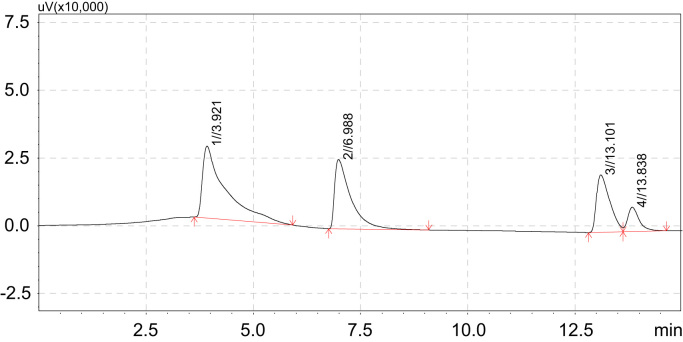
Sample standard.

**Fig. A17:**
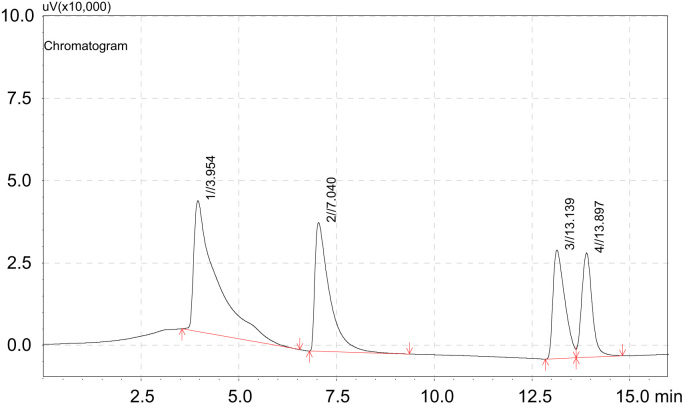
Sample standard.

**Fig. A18:**
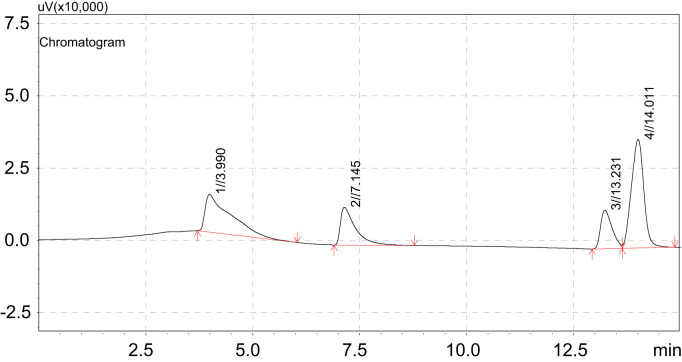
Sample standard.

**Fig. A19:**
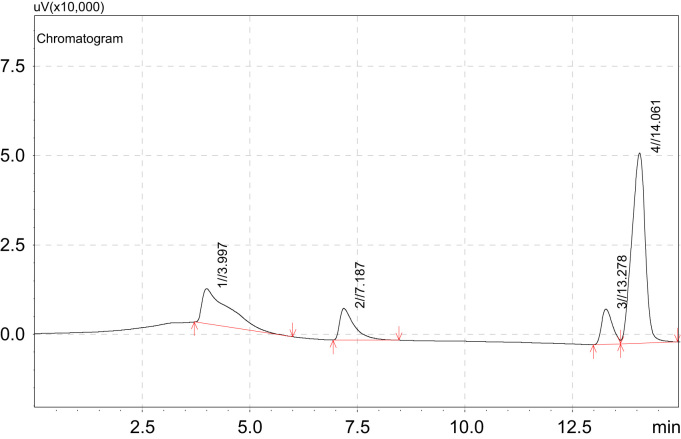
Sample standard.

**Fig. A20:**
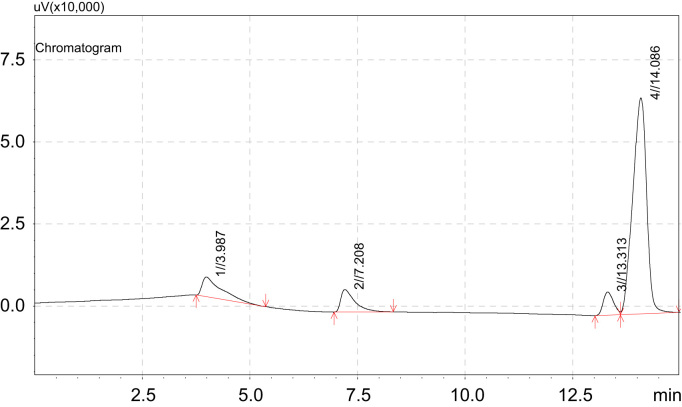
Sample standard.

These standards are injected manually first into GC column using the micro syringe. Each sample is injected 3–5 times and observed chromatogram for the analysis. A chromatogram of the each sample standard is shown in Fig. A1, Fig. A2, Fig.A3, Fig. A4, Fig. A5, Fig. A6, Fig. A7, Fig. A8, Fig. A9, Fig.A10, Fig. A11, Fig. A12, Fig. A13, Fig. A14, Fig. A15, Fig. A16, Fig. A17, Fig. A18, Fig. A19, Fig. A20–Fig. A1, Fig. A2, Fig.A3, Fig. A4, Fig. A5, Fig. A6, Fig. A7, Fig. A8, Fig. A9, Fig.A10, Fig. A11, Fig. A12, Fig. A13, Fig. A14, Fig. A15, Fig. A16, Fig. A17, Fig. A18, Fig. A19, Fig. A20. The order of the peaks observed from Fig. A1, Fig. A2, Fig.A3, Fig. A4, Fig. A5, Fig. A6, Fig. A7, Fig. A8, Fig. A9, Fig.A10, Fig. A11, Fig. A12, Fig. A13, Fig. A14, Fig. A15, Fig. A16, Fig. A17, Fig. A18, Fig. A19, Fig. A20–Fig. A1, Fig. A2, Fig.A3, Fig. A4, Fig. A5, Fig. A6, Fig. A7, Fig. A8, Fig. A9, Fig.A10, Fig. A11, Fig. A12, Fig. A13, Fig. A14, Fig. A15, Fig. A16, Fig. A17, Fig. A18, Fig. A19, Fig. A20 are, first water peak obtained at 4 min retention time, second methanol peak at 7 min, third methyl acetate at 13.6 min and acetic acid at 13.9 min. The exact starting point of the depending on its concentration. The methyl acetate peak contacting with acetic acid peaks. Several strategies were tried to get better peak shape. The possibilities we tried to get better shape of the peak are changing the liner, changing the polarity, use of different carrier gas, cleaning the injection line, hanging the flow rate of the carrier gas, adjusting the column and oven temperatures and current. Here the best chromatograms obtained in our experimental conditions are presented in Fig. A1, Fig. A2, Fig.A3, Fig. A4, Fig. A5, Fig. A6, Fig. A7, Fig. A8, Fig. A9, Fig.A10, Fig. A11, Fig. A12, Fig. A13, Fig. A14, Fig. A15, Fig. A16, Fig. A17, Fig. A18, Fig. A19, Fig. A20–Fig. A1, Fig. A2, Fig.A3, Fig. A4, Fig. A5, Fig. A6, Fig. A7, Fig. A8, Fig. A9, Fig.A10, Fig. A11, Fig. A12, Fig. A13, Fig. A14, Fig. A15, Fig. A16, Fig. A17, Fig. A18, Fig. A19, Fig. A20.

### 2.6 Calibration curves

All the samples were injected to GC column to analyze composition of the known component mixture. The sample volumes are maintained constant for all the experimental runs. In the present experimental analysis the sample volume of 1 µl is taken to avoid variation of the peak sizes. While considering the peak area instead of area percentages of the peaks it is giving errors. The reason is that when the small size samples are injected instead of the constant sample volume, the area of the peaks differs but the percentage of the peaks always constant. Because the peak area is directly proportional to the amount the liquid injected into the column. When the small sample volume injected, the diminishing of all the peaks occur proportionally. That means the area percentage is constant for small or large size samples but total area is not constant. But the composition is same which is independent of the size of the sample volumes. The samples are injected into the column in a batch operation.

The integration of the chromatograph was made by the GC Solutions software supplied by Shimadzu Company. A sample chromatogram is displayed in Fig. 3.

The area percentage of components plotted against composition (mass fraction or the molar fraction) is shown in Fig. 4. It is found that the plot of area versus mole faction gives the better linear aspect than the area versus mass fraction plot. So the calibration is done based on the mole fraction instead of the mass fraction.

## Appendix A

Fig. A1, Fig. A2, Fig.A3, Fig. A4, Fig. A5, Fig. A6, Fig. A7, Fig. A8, Fig. A9, Fig.A10, Fig. A11, Fig. A12, Fig. A13, Fig. A14, Fig. A15, Fig. A16, Fig. A17, Fig. A18, Fig. A19, Fig. A20–Fig. A1, Fig. A2, Fig.A3, Fig. A4, Fig. A5, Fig. A6, Fig. A7, Fig. A8, Fig. A9, Fig.A10, Fig. A11, Fig. A12, Fig. A13, Fig. A14, Fig. A15, Fig. A16, Fig. A17, Fig. A18, Fig. A19, Fig. A20. Characteristic peaks of a Gas Chromatograph for the four component system of Acetic acid–Methanol–Methyl Acetate–Water for different concentrations as given in Table 1 (The figures as given from A.1–1.20).
