# Supplementary material for: Data on acetic acid–methanol–methyl acetate–water mixture analysised by dual packed column Gas Chromatography
Source: Data Brief. 2018 Mar 29;18:947–60. doi: 10.1016/j.dib.2018.03.111 (PMC5996402; doi:10.1016/j.dib.2018.03.111)
Supplement: Supplementary file 1 — Supplementary material [file mmc1.zip › Conflicts of Interest.pdf]

Conflicts of Interest

The authors have to declare that no conflicts of interest.
